# Potential implications of the climate crisis on diagnostics

**DOI:** 10.1371/journal.pgph.0002935

**Published:** 2024-03-08

**Authors:** Bernard Owusu Agyare, Paul Eder, Shubhada Shenai, Bih H. Chendi, Colin Carlson, Ange Iradukunda, Marithe Mukoka, Jack Ogony, Olivier Manigart, Ephraim Ogbaini-Emovon, Ahmed A. Seida, Esteban O. Prado, Anisa Ghadrshenas, Sergio Carmona, Joseph D. Tucker

**Affiliations:** 1 Center for Global Health Science and Security, Georgetown University, Washington, DC, United States of America; 2 National Institute of Allergy and Infectious Diseases, National Institutes of Health, Bethesda, Maryland, United States of America; 3 FIND, New Delhi, India; 4 Institute of Clinical Medicine, Faculty of Medicine, University of Oslo, Oslo, Norway; 5 SAMRC Centre for Tuberculosis Research, Division of Immunology, Department of Biomedical Sciences, Stellenbosch University, Cape Town, South Africa; 6 Heart to Heart International, Kigali City, Rwanda; 7 Rodolphe Meriuex Laboratory INRB- Goma, Goma, Democratic Republic of Congo; 8 College of Health Sciences, Jomo Kenyatta University of Agriculture and Technology, Nairobi, Kenya; 9 GFA Consulting Group, Bobo-Dioulasso, Burkina Faso; 10 PROALAB, West African Health Organization, Bobo-Dioulasso, Burkina Faso; 11 Institute of Lassa Fever Research and Control, ISTH, Irrua, Edo, Nigeria; 12 Immunology and Microbiology Department, Faculty of Veterinary Medicine, Cairo University, Cairo, Egypt; 13 University of Las Américas (UDLA), Quito, Equador; 14 UNITAID, Geneva, Switzerland; 15 FIND, Geneva, Switzerland; 16 Clinical Research Department, Faculty of Infectious and Tropical Diseases, London School of Hygiene and Tropical Medicine, London, United Kingdom; 17 Institute for Global Health and Infectious Diseases, University of North Carolina at Chapel Hill, Chapel Hill, North Carolina, United States of America; PLOS: Public Library of Science, UNITED STATES; McGill University, CANADA

Many countries, especially low- and middle-income countries (LMICs), have not planned for how the climate crisis will increase the need for diagnostics, even as environmental changes are accelerating expansion of human and animal disease [1]. Diagnostics for human diseases are essential for anticipating and responding to health impacts of the climate crisis and play a fundamental role in identifying new infectious disease outbreaks, informing research and predictive models, monitoring the effectiveness of interventions, and spurring public health policy in diverse global settings. Enhanced human diagnostics will be important for public health, veterinary, public health, and environmental sectors. This Opinion uses a One Health approach to highlight the potential implications of the climate crisis on diagnostics and priorities for adaptation.

**Fig 1:**
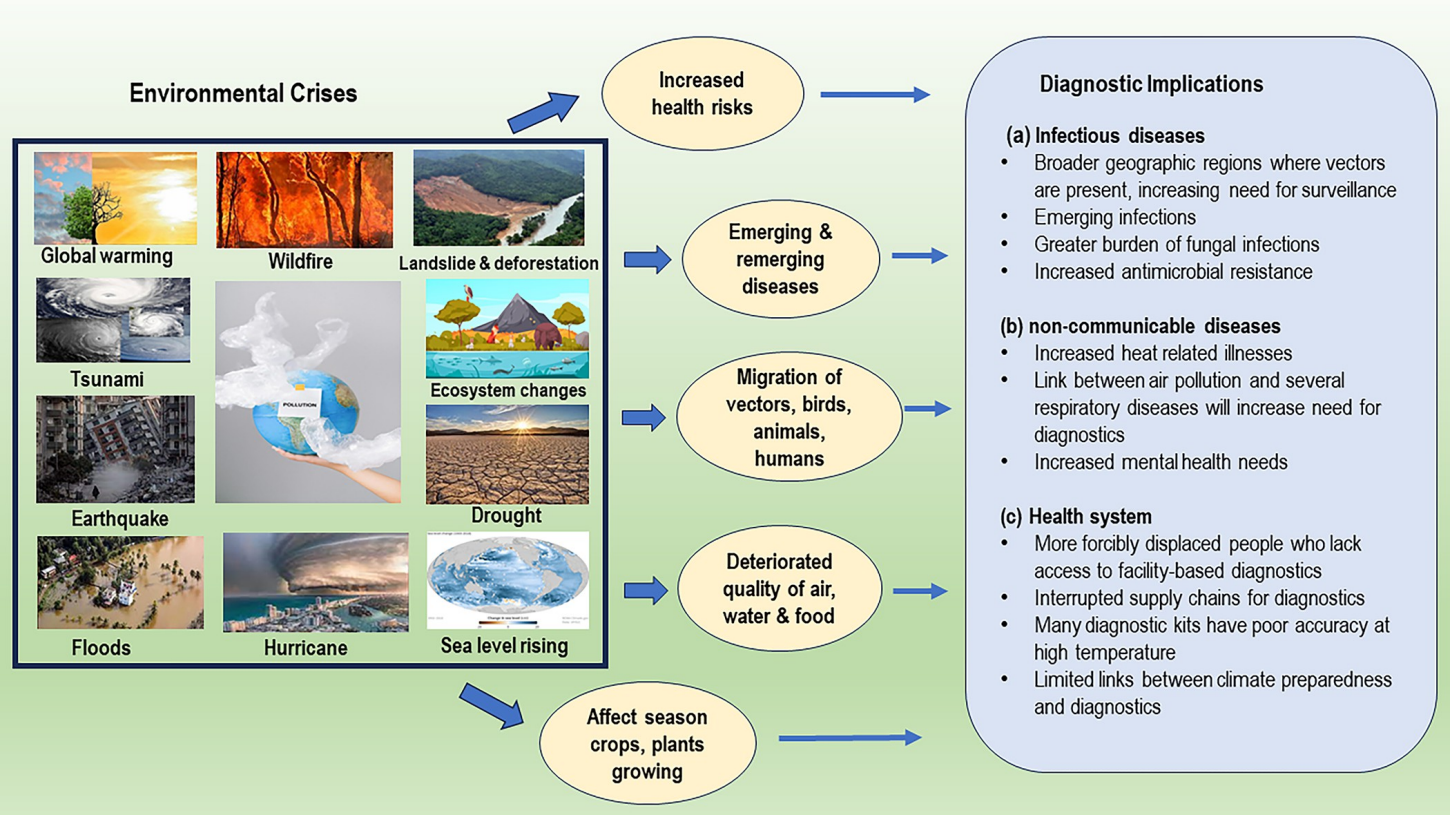
Environmental crises from climate change and implications for diagnostics. Image of a tsunami, retrieved from Wikimedia Commons (https://commons.wikimedia.org/w/index.php?search=hurricane&title=Special:MediaSearch&go=Go&type=image&haslicense=unrestricted); image of an ecosystem, retrieved from Freepik (https://www.freepik.com/free-vector/biological-hierarchy-cartoon-colorful-demonstrated-ecosystem-with-plants-animals-fishes-illustration_16396079.htm#query=ecosystems&position=0&from_view=keyword&track=sph&uuid=abeaf967-f5f2-4c55-b4ec-fc9b46440f15); image of a globe illustrating pollution, retrieved from Pexels (https://www.pexels.com/photo/blue-globe-with-plastic-4167579/); image of a sea level rise, retrieved from Wiki Commons (https://commons.wikimedia.org/wiki/File:Sea_level_change_1993_to_2018.jpg).

The climate crisis has several potential implications on diagnostics not only for infectious diseases, but also non-communicable diseases (NCDs) and entire health systems (Fig 1). From an infectious disease perspective, increased vector-borne disease (e.g., dengue, malaria, Crimean-Congo hemorrhagic fever virus) transmission from insect- and other carrier-migration to regions previously unexposed will require broader surveillance as well as diagnostic testing capabilities [2]. For example, Burkina Faso has experienced increasingly long rainy seasons, leading to a broader geographic penetration of larval breeding sites for *Aedes aegypti* and *Ae*. *albopictus* species. Coincident with increased larval breeding is increased dengue disease. In 2023, there were more than 79,000 suspected dengue cases and nearly 350 deaths. Yet dengue diagnosis in Burkina Faso remains restricted to symptoms alone because molecular diagnostic testing (e.g., PCR) is not widely available.

In addition to vector-borne spread of viral diseases, invasive fungal infections from rising global temperatures and broader water-related disasters (floods, hurricanes, mudslides) are a serious threat to millions of individuals around the globe, resulting in more than 1.5 million deaths annually [2]. These infections are a global health issue, especially for critically ill patients in hospitals, patients with compromised immune systems, and older adults. Despite invasive fungal infections becoming more common (e.g. cocciodiodies cases [3]), there are few approved fungal diagnostic tests broadly available. Fungal diagnostic tests have not been prioritized by funders, public health agencies, or researchers. Last, antimicrobial resistance from otherwise common bacterial infections is increasing in many global community settings, with some evidence that climate change is contributing to this global health problem [4]. These trends will have implications for infectious disease diagnosis especially in LMICs where diagnostic capacity is already weak because of fewer laboratory personnel and less state-of-the-art equipment.

The climate crisis will also influence diagnostics for non-communicable diseases. For example, burgeoning air pollution will increase the risk of respiratory disease, such as chronic obstructive pulmonary disease (COPD) [5]. This problem will be borne largely by the poor who are more likely to live in areas with unsafe levels of air pollution [6]. Air pollution from industrial output exacerbates climate change conditions, because industrial-produced chemical species that decrease air quality are often co-emitted with greenhouse gases [7]. This will increase the need for diagnostics that can differentiate, for example, COPD from syndromes that are caused by human-induced air pollutants that contribute to the climate crisis.

Other examples of non-communicable diseases affected by climate change include mental health conditions. The increased trauma due to forced human migration, living day-to-day among toxic environmental ground and water conditions, long-term care of disease-stricken family members can all take a mental toll on individuals and families [8]. Further consideration of how best to diagnose mental health disorders in pre, during, and post-migration settings [9] will be essential as part of climate crisis preparedness and adaptation [10].

Beyond climate change impacts on individual disease diagnostics, entire community health diagnostic systems are also impacted by climate change. The climate crisis will increase the number of forcibly displaced people because of home destruction, uncertainty, and economic vulnerability. The United Nations Refugee Agency has estimated that 21.5 million people are already displaced by weather-related disasters each year [11]. The worst-affected populations during displacements are often socially and economically vulnerable groups who lack access to health care, including diagnostics. Mass population displacement will challenge conventional diagnostic services that require people to reach hospitals. Many migrants are excluded from local diagnostic services [12]. In addition, floods, fires, and other natural disasters will interrupt diagnostic supply chains and decrease the availability of raw materials necessary for several diagnostics. Changes in temperature and humidity often affect the stability and performance of diagnostic kits, reagents, and laboratory equipment [13].

Given the challenges and pace of the climate crisis, diagnostics must rapidly adapt. Expanded diagnostic services will be needed for vector-borne and fungal diseases, especially in areas with limited capacity now, such as lower level health facilities. Accelerated regulatory pathways, coordinated field testing, and disease-agnostic platforms will be crucial in preparing and responding to future epidemics. Emergency response in public health disaster and post-disaster settings will benefit from decentralized diagnostics (self-testing and related self-care strategies) that require no laboratory infrastructure [14].

Health systems responses to ensure access for all to diagnostics will be essential for climate adaptation efforts. Building capacity for remote diagnostics (including telemedicine), self-care approaches (e.g., self-testing, self-sampling), and regional manufacturing capacity can sustain diagnostic access during disasters. Given alignment with vaccine priorities, collaboration between diagnostics and vaccine groups may accelerate advocacy. Mass deployment of diagnostics in emergency settings will be vital to swiftly address and manage climate-related health crises. The climate crisis response must address social dimensions of diagnostic access, reaching climate refugees are likely to disproportionately bear the burden of climate change. Community engagement, including product design with end-users, will help to decrease some of the pervasive disparities that exist in diagnostics. Developing diagnostic test kits that reduce carbon emissions is also important, as are measures to reduce plastic and other waste generated by discarded tests. Deployment of diagnostics must be integrated into disaster preparedness simulations. This will enhance our ability to accurately anticipate short-term impacts of climate-related disasters and can help disaster response teams better appreciate the importance of diagnostics in preparedness and response.

The climate crisis will have important implications for people who develop, use, and rely on diagnostics. For clinicians and public health practitioners, the emergence of novel pathogens, re-emergence of some infectious diseases, and increased incidence of NCDs will require increased support for diagnostics, quality assurance, and enhanced surveillance. For diagnostic manufacturers, developing diagnostics with less carbon footprint and adaptation strategies for environmental disasters are critical next steps. Policy makers involved in developing climate action plans at the global, national, and subnational levels should consider diagnostic access when assessing preparedness and planning activities (e.g., setting national adaptation goals under the Paris Agreement). Finally, the climate crisis necessitates a new research agenda that captures human, animal, agricultural and environmental dimensions of health. Embracing this One Health approach could help to articulate climate crisis impacts in these areas and develop responses.
